# The Mediation Role of the Risk of Non-Alcoholic Fatty Liver Disease in Relationship between Lutein and Zeaxanthin and Cognitive Functions among Older Adults in the United States

**DOI:** 10.3390/nu14030578

**Published:** 2022-01-28

**Authors:** Chen Chen, Zhonghai Lu, Dongfeng Zhang, Suyun Li

**Affiliations:** Department of Epidemiology and Health Statistics, School of Public Health, Qingdao University, No. 308, Ningxia Road, Qingdao 266021, China; 2020021065@qdu.edu.cn (C.C.); 2020021086@qdu.edu.cn (Z.L.); zhangdongfeng@qdu.edu.cn (D.Z.)

**Keywords:** lutein and zeaxanthin, non-alcoholic fatty liver disease, cognition, NHANES, mediation analysis

## Abstract

Background: Previous studies showed lutein and zeaxanthin (L and Z) may influence cognitive function by different mechanisms. Our study aimed to be the first to examine whether the risk of non-alcoholic fatty liver disease (NAFLD) mediated the possible association between the dietary intake of L and Z and cognitive function. Methods: We conducted a cross-sectional analysis of participants aged 60 years or over in the National Health and Nutrition Examination Survey (NHANES) 2011–2014. Multivariable linear regression was used to investigate the association between the dietary intake of L and Z and cognitive function, and structural equation modeling tested the mediation effect. Results: The fatty liver index for the United States population (US FLI) acted as a mediator in the association between the higher intake of L and Z and the Animal Fluency Test, the Digit Symbol Substitution Test (DSST), and composite score and mediated 13.89%, 17.87%, and 13.79% of the total association in dietary L and Z intake (14.29%, 13.68%, and 10.34% of the total association in total L and Z intake), respectively. Conclusion: Our study indicated the potential role of the risk of NAFLD as a mediator of associations between the dietary intake of L and Z and cognitive function in the geriatric American population.

## 1. Introduction

With the increase in the number of older population members worldwide, the health problems of older adults are becoming increasingly serious. In particular, aging may lead to age-related cognitive impairment. Owing to vascular risk factors, cardiovascular health declines with age, and this causes an increase in the risk of cognitive impairment [1]. Mild cognitive impairment (MCI) is known as the negative and pathological cognitive changes beyond what is expected in normal aging and is a potential transitional process between normal aging and dementia (mainly Alzheimer’s disease (AD)) [1]. Cognitive impairment continues to worsen without any prevention or intervention and develops into pathological MCI and even dementia [2]. Previous study showed that an estimated 35.6 million people worldwide had dementia in 2010, compared with an estimated 65.7 million in 2030 and 115.4 million in 2050 [3]. Cognitive health has become an important public health issue affecting the aging population all over the world. The progression of cognitive impairment to AD is irreversible, and there are numerous cases of treatment failure. There is currently no cure for AD, so it is necessary to identify changeable factors that affect cognitive function and to intervene before older adults develop AD.

A cohort study indicated that dietary antioxidant deficiency is a modifiable risk factor for cognitive decline [4]. Carotenoids are dietary antioxidants; lutein and zeaxanthin (L and Z hereafter) are 2 of 600 natural carotenoids, and they must be obtained by diet, mainly through the intake of green leafy vegetables and varieties of fruits [5]. L and Z are, structurally, isomers of each other, and it is difficult to distinguish L and Z. Previous studies have always reported them as a class of substances, the most notable feature of which being that they preferentially accumulate in the macula of the eye [6] and brain [7], including frontal, occipital, and temporal cortices, cerebellum, and pons. Above all, their levels in the brain account for 66–77% of the total brain carotenoid levels [5]. In previous literature involving United States (US) older samples, L and Z were positively correlated with multiple measures of cognitive functions [8,9,10]. Although some studies have shown that L and Z may influence cognition through anti-inflammatory and antioxidant properties, the underlying mechanisms are not clear. Moreover, the analysis of the mediation between L and Z, and cognition is also limited. To sum up, it is necessary to explore the mechanism between L and Z and human cognitive functions.

The anti-inflammatory and antioxidant capacities of L and Z may also affect the inflammatory development of non-alcoholic fatty liver disease (NAFLD) [11] and may also hinder the metabolism of lipids, thus aggravating fatty liver disease. NAFLD is the most common type of chronic liver disease in the US, and its incidence is on the rise globally [12]. Epidemiological studies and laboratory animal studies have found significant associations between a higher carotenoid intake (including L and Z) and a lower risk of NAFLD [13,14,15]. Existing evidence has shown that treatment with lutein lowers the levels of inflammatory genes in the body [16].

Moreover, complications of NAFLD have been reported to include cognitive impairment [17,18]. Up to 70% of NAFLD cases are associated with a negative effect on cognitive problems, such as memory and attention [19]. NAFLD was recently identified as metabolic-associated fatty liver disease (MAFLD) [20] and as an independent risk factor acting on vascular dysfunction and cognitive-related disorders [18]. Therefore, we speculate that the risk of NAFLD may have a mediating effect between L and Z intake and cognitive functions.

No previous study has directly explored whether the risk of NAFLD mediates dietary L and Z intake and cognitive functions in older adults. Therefore, we explored the mediation role of the risk of NAFLD and explain the details of the pathway. The objective of our study was to investigate the existence of mediating effects, with the possibility that the risk of NAFLD could be an important intervention target to intervene upon with the decline in cognition in older adults.

## 2. Materials and Methods

### 2.1. Study Design

The National Health and Nutrition Examination Survey (NHANES) is a cross-sectional survey designed to provide a representative sample of the US non-institutionalized civilian population [21]. The National Center for Health Statistics Ethics Review Board of Centers for Disease Control (CDC) sanctioned all NHANES projects and every participant signed their informed consent.

Two cycles (2011/2012 and 2013/2014) included the collected information via cognitive function tests. A total of 19,931 participants took part in the 2011–2014 NHANES. Among the participants aged 60 years or over (*n* = 3632), we excluded participants based on the following criteria: missing information about the fatty liver index for the US population (US FLI), cognitive tests, L and Z dietary intake, and covariates (*n* = 2423); individuals characterized by the production of hepatitis B or C antibody (*n* = 128); and missing information about alcohol intake or excessive alcohol drinking (1 drink/day for women or 2 drinks/day for men) (*n* = 453). The participants who had other self-reported liver diseases (*n* = 24) were also excluded to avoid the confounding effects. Finally, 604 participants (weighted *n* =29,397,912) were included in the analysis.

### 2.2. Lutein and Zeaxanthin in Dietary and Total Intake

In total, 2 sets of 24 h dietary information were obtained via interviews in the 2 cycles; they were used to assess the participants’ dietary nutrients and energy intake during these periods. The interviews were conducted in a private room in the Mobile Examination Center (MEC), containing a standard set of measuring guides. The first dietary recall was collected in-person during the NHANES visit, while the second was collected over telephone 3–10 days later. After the first dietary recall interview, the participants were given measuring cups, spoons, a ruler, and two-dimensional drawings of the various measuring guides available in the MEC, to use for reporting food amounts during the telephone interview [22]. The total estimated dietary L and Z intake was averaged over the two recall periods (if only the first day was available, that value was used instead of the average). The total energy intake was also acquired during the 24 h dietary recall. The participants were queried about supplement use for the same two 24 h periods; L and Z intake from supplements was also averaged over two days if available. The total L and Z intake was calculated as the sum of dietary and supplement intake.

### 2.3. NAFLD—Related Measurement

Patients with NAFLD present with excessive deposition of fat in the liver, different from other liver diseases due to other causes and too much alcohol intake. The prediction of the risk of NAFLD was based upon the US FLI [23,24,25]. The fatty liver index (FLI) was first found from a study in north-central Italy. Then, the US FLI was derived for the multi-ethnic US NHANES and was used in healthy people to estimate the risk of NAFLD [24]. The specific formula is as follows.

(1) $$\mathrm{US}\;\mathrm{FLI}=\left(e^{-0.8073\times \mathrm{non}-\mathrm{Hispanicblack}+0.3458\times \mathrm{MexicanAmerican}+0.0093\times \mathrm{age}+0.6151{\times \log}_{e}\left(\mathrm{GGT}\right)+0.0249\times \mathrm{waistcircumference}+1.1792{\times \log}_{e}\left(\mathrm{insulin}\right)+0.8242{\times \log}_{e}\left(\mathrm{glucose}\right)-14.7812}\right)/\;\;(1+e^{-0.8073\times \mathrm{non}-\mathrm{Hispanicblack}+0.3458\times \mathrm{MexicanAmerican}+0.0093\times \mathrm{age}+0.6151{\times \log}_{e}\left(\mathrm{GGT}\right)+0.0249\times \mathrm{waistcircumference}+1.1792{\times \log}_{e}\left(\mathrm{insulin}\right)+0.8242{\times \log}_{e}\left(\mathrm{glucose}\right)-14.7812})\times 100.$$

The US FLI ranges from 0 to 100 [24]. In our analyses, we used the US FLI as a continuity variable to explore the importance of early dietary prevention of NAFLD on cognitive functions. 

### 2.4. Cognitive Outcomes

In the cycles of NHANES (2011–2014), cognitive function tests were carried out at the MEC for participants aged 60 years or older [26,27]. Three tests were included—namely, the Consortium to Establish a Registry for AD Word Learning subtest (CERAD W-L), the Animal Fluency Test, and the Digit-Symbol Substitution Test (DSST). The CERAD W-L executed the task of immediate and delayed recall of new verbal information consisting of three consecutive learning trials, as well as a delayed recall. For each test, the participants were asked to read 10 words and then recall as many learned words as they could immediately and after a few minutes. In the end, the results were calculated by the number of right answers as three individual trial scores ranging from 0 to 10, a total score among all three trials ranging from 0 to 30, and one delayed recall score ranging from 0 to 10. The Animal Fluency Test was used to evaluate categorical verbal fluency (a vital component of executive functions). Participants were instructed to list as many animal names as they could in one minute and obtained one point when a correct answer was given. Although there was no upper limit, the total score ranged from 3 to 36 in our analysis. The DSST, a module of the Wechsler Adult Intelligence Test, was used to evaluate processing speed, sustained attention, and working memory, and its score ranged from 2 to 105 in our analysis. Each participant was provided a piece of paper and was required to match the right symbols in 133 boxes with numbers in 2 min and connect them. The total score was the number of correct matches. Then, the Z-score was calculated from the average of the total standardized scores on the three cognitive tests to estimate the overall score level.

### 2.5. Statistical Analysis

All statistical analyses were adjusted for the survey design and the weighting of the variables to illustrate the complex sampling design, making sure of national representation. Fasting information in this study was collected from those sample participants who were subsampled to fast before attending Mobile Examination Center (MEC) exams. Due to the smallest subpopulation being in the fasting group, we used the fasting subsample weights (wtsaf4yr). Because of the two cycles included, we created four-year new weights as one-half of the value of the fasting sub-sample MEC weight (WTSAF2YR×1/2) included in all analyses, according to the analytical guidelines of the NHANES [28]. The characteristics across the different groups are presented as mean ± standard error (SE) for continuous variables and as proportion (SE) for categorical variables. The US FLI and cognitive function tests were compared among different L and Z groups using a one-way analysis of variance (ANOVA). To examine the relation of L and Z intake to US FLI and cognitive function scores, L and Z intake was divided into four groups according to the quartiles based on frequency distributions (quartiles (Q) of L and Z intake; L and Z dietary intake ranged from 0 to 532 mcg, from 532.5 to 900 mcg, from 901.5 to 1801.5 mcg, and from 1816.5 to 146911.5 mcg in Q1, Q2, Q3, and Q4, respectively. Moreover, L and Z total intake ranged from 0 to 569.5 mcg, from 578.5 to 1011 mcg, from 1022.5 to 2055 mcg, and from 2076.5 to 161911.5 mcg in Q1, Q2, Q3, and Q4, respectively). The correlations among cognitive composite scores, US FLI, and dietary or total L and Z intake were investigated by conducting multiple linear regressions. Mediation analyses were used to investigate whether the US FLI significantly influenced the relationship between the L and Z groups (Q2 vs. Q1, Q3 vs. Q1, and Q4 vs. Q1) and cognitive function tests. Then, we transformed four categories of L and Z intake into three dummy codes and tested the mediation by structural equation modeling (SEM), as suggested by Hayes and Preacher (2014), combining bootstrapping sampling methods and bias-corrected bootstrap confidence intervals [29]. The relative direct effect and indirect effect of groups across US FLIs were analyzed. Age, gender, race/ethnicity, educational level, total energy intake, and the survey year of the participants were included as covariates. All the statistical analyses were conducted using Stata SE 15.0 (Stata Corp LP, College Station, TX, USA). A *p*-value < 0.05 was used to define statistical significance, and all tests were two-sided.

## 3. Results

### 3.1. General Characteristics

Table 1 shows the basic characteristics’ comparison between the two NHANES cycles’ participants aged 60 years or over and the participants involved in our analysis. We included 604 participants in our study and carried out the weighted process (weighted *n* = 29, 397, 912). When classified by NHANES cycles and sex, the participants’ distribution was relatively even, just as was found by previous research studies in the same area [30]. The mean age of the participants was about 68–70 years. In terms of racial proportion, the majority self-reported as non-Hispanic white (about 87%), followed by non-Hispanic Black (around 5%). Moreover, there were approximately 88% participants having undergone education for more than 12 years. The mean value of the components involved in the US FLI and the scores of the three cognitive tests were generally higher in the analyzed group. The three cognitive function tests, Z-score, and US FLI were all associated with dietary and total L and Z intake (Table 2). The differences among the four cognitive indexes and the US FLI in the four groups of L and Z intake were statistically significant.

### 3.2. Association of Dietary L and Z Intake with Cognitive Functions

In Table 3, the comparisons between the crude model and adjusted model show the change in and 95% confidence interval of the cognitive function scores with the per-quartile increase in dietary or total L and Z intake. After adjusting for confounders, no matter the dietary or total intake, the Q4 group of L and Z was positively associated with three cognitive function scores and Z-score compared with the first group. In the model of total intake of L and Z, both the Q3 and Q4 groups of L and Z showed significant associations, suggesting increased intake of L and Z may be relevant to higher cognitive test scores or satisfactory performance.

### 3.3. Association of the US FLI with Cognitive Functions

As Table 3 shows, the association between the US FLI and cognitive functions was statistically significant. In the adjusted model, a lower US FLI was related to higher cognition test scores and Z-score, with the β(95% CI) being −0.03 (−0.06, −0.01), −0.09 (−0.16, −001), and −0.004 (−0.01, −0.00) for the Animal Fluency Test, DSST, and Z-score, respectively.

### 3.4. Association of Dietary L and Z Intake with US FLI

Table 4 shows us that a higher L and Z intake may correlate with a lower US FLI. The Q4 group of dietary L and Z intake, compared with the control group, was negatively associated with the US FLI (β= −10.23); the results of the Q4 group of L and Z total intake results were similar to the results of L and Z dietary intake (β= −9.84) after adjusting the confounding factors.

### 3.5. Mediation Effects of the US FLI on Relationship between Dietary or Total L and Z Intake and Cognitive Functions

**Figure 1:**
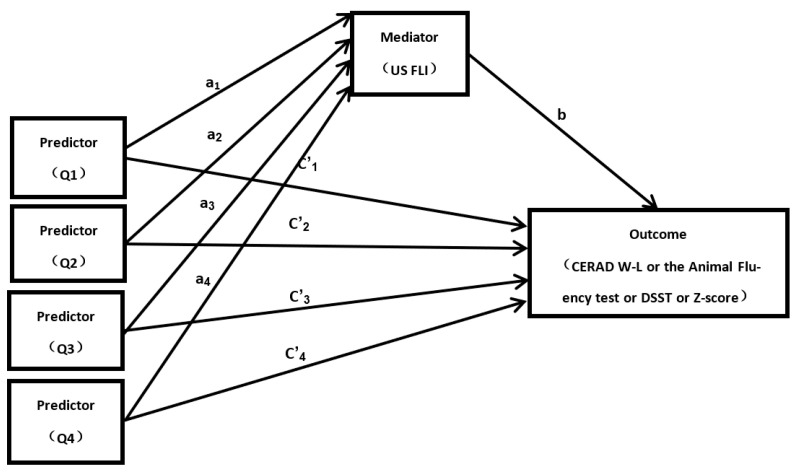
Path diagram of mediation model. “a_1_–a_4_”: the effect of per category of dietary or total L and Z intake on the US FLI; “**b**”: the effect of US FLI on cognitive function (CERAD W-L or the Animal Fluency test or DSST); “**c’_1_**–**c’_4_**”: the direct effect of per category of dietary or total L and Z intake on cognitive function (CERAD W-L or the Animal Fluency test or DSST). The first category of dietary or total L and Z intake is the reference group. Original to this manuscript.

Figure 1 provides a path model that indicates the mediation effect of the US FLI. In Table 5 and Table 6, for the relative mediation effect analysis, the results of the bootstrapping show that the US FLI had a significant relative indirect effect on both the dietary and total L and Z intake Q4 group and Animal Fluency Test score (indirect effect (a_n_ × b): 0.30), DSST score (indirect effect (a_n_ × b): 0.84), and Z-score (indirect effect (a_n_ × b): 0.04). The results of the bootstrapping also show that the US FLI had a significant relative indirect effect on the total L and Z intake Q4 group and the Animal Fluency Test score (indirect effect (a_n_vb): 0.30), DSST score (indirect effect (a_n_ × b): 0.78), and Z-score (indirect effect (a_n_×b): 0.03). Compared with the dietary control group (Q1), the Q4 group had increased Animal Fluency Test score, DSST score, and Z-score by 0.30, 0.84, and 0.04 units, respectively. Compared with the total intake control group (Q1), the Q4 group had increased Animal Fluency Test score, DSST score, and Z-score by 0.30, 0.78, and 0.03 units, respectively. In Table 6, we can see that the direct effects of the total L and Z intake Q4 group on the four cognitive function scores were all significant. All the results above indicate that the US FLI mediated the relationship between higher L and Z intake and cognitive functions. As for the mediation analysis from the bias-corrected bootstrap, the proportion of the US FLI explaining the dietary L and Z intake Q4 group was 13.89% for the Animal Fluency Test, 17.87% for the DSST, and 13.79% for the Z-score. The proportion of the US FLI explaining the total L and Z intake Q4 group was 14.29% for the Animal Fluency Test, 13.68% for the DSST, and 10.34% for the Z-score. Higher L and Z intake may be indirectly related to better cognitive performance among the factors of reduced risk of NAFLD.

## 4. Discussion

This cross-sectional study used two NHANES cycles (2011–2012 and 2013–2014) and explored whether the risk of NAFLD mediated the relationship between dietary L and Z intake and cognitive functions in older adults in the US. The link between the risk of NAFLD and cognitive functions was analyzed in the continuous NHANES database for the first time.

### 4.1. Association between Dietary L and Z Intake with Cognitive Functions

The higher the L and Z intake was, the better cognitive performance in the memory, language, and executive function fields was. The results of this study are broadly consistent with those of previous studies in populations at risk of NAFLD [5,30,31,32,33,34]. After adjusting for potential confounders, we found that the relationships between dietary L and Z intake and three cognitive tests’ scores remained statistically significant. Studies have shown that a moderate intake of L and Z is a sign of a healthy lifestyle and may reduce oxidation or inflammation and maintain meningeal stability [33,35]. Additionally, L and Z accumulate in the membranes of brain tissues and have protective effects on the axons, which play a significant role in the structural and functional integrity of the membranes of brain cells [36]. Similarly, cognitive impairment is associated with brain tissue damage, dysfunction, cell death, and cell and nerve junction damage. Such evidence provides an important basis for the study of the relationship between dietary L and Z intake and cognitive functions.

### 4.2. Dietary L and Z Intake, NAFLD and Cognitive Functions

This analysis used the US FLI to assess the risk of NAFLD. The US FLI is a simple, non-invasive scoring system measuring the risk of fatty liver in people in the US. Our study found that dietary and total L and Z intake were related to the US FLI and so were the relationships between the US FLI and three cognitive tests (including Z-scores).

A study of US adults showed that the higher levels of L and Z carotenoid intake may reduce the risk of NAFLD [37] and inhibit the progression of simple hepatic steatosis to nonalcoholic steatohepatitis (NASH) by reducing oxidative stress responses in the liver and pro-inflammatory cytokines secreted by liver macrophages, as well as by reducing immune infiltration and insulin sensitivity [11]. Furthermore, animal studies have supported that carotenoid (including lutein) administration may impede the accumulation of lipids in the liver with the consumption of a high-fat diet [15], increasing the expression of SIRT1, a key gene regulator for fatty acid oxidation. Kim’s animal tests on guinea pigs showed that lutein supplementation could lower the factor levels in guinea pigs on a high-cholesterol diet by reducing the DNA binding activity of NF-κB [38]. Qiu’s experiment found that lutein supplementation reduced the hepatic TC and TG levels in hyperlipidemia rats and increased the levels of PPAR-αprotein, thereby enhancing the oxidation of fatty acids [15,39]. In addition, the US FLI was found to be higher in people with lower L and Z intake, indicating a greater risk of NAFLD. In summary, carotenoids may influence hepatic disease status by disturbing lipid metabolism. On the other hand, a review has shown that NAFLD is a multi-system disease that may influence cognition through systemic and neuro-inflammation, metabolic liver dysfunction and ammonia metabolism disorder, disturbed gut microbiota, atherosclerosis and cerebrovascular dysfunction, neurodegeneration, and obstructive sleep apnea [40].

The above mechanisms provide evidence of the existence of the mediation effects. The finding indicating that the risk of NAFLD mediated the association between L and Z and cognitive functions further underscores the crucial role of the risk of NAFLD in the development of cognitive impairment in individuals under L and Z intake treatment. Thus, the results further provide the recommendation for the risk assessment of NAFLD and early L and Z supplementation to reduce the detrimental effects of cognitive impairment.

### 4.3. Study Strengths and Limitations

There are several strengths in our study. First, the main strength is the use of the NHANES survey, which is of high quality due to the survey methods and quality control. Second, the validity of the results is explained by the fact that the mediating effect of the US FLI still existed after adjusting for major confounders. Third, our study is the first to test the relationship between cognition and the US FLI in the continuous NHANES database. Fourth, to estimate the risk of NAFLD, our study used the US FLI—a better, non-invasive system index, compared to an invasive liver biopsy. Additionally, the use of SEM for the mediation analysis of multiple classification-independent variables also has advantages: the measurement error could be effectively controlled to obtain a more accurate mediation effect value [29], and the bootstrap method was integrated into the analysis of SEM.

There are also some limitations in our study. First, this was a cross-sectional study. Further prospective studies are needed to collect more unmeasured confounders to verify the mediating effect. Second, the supplement information for dietary L and Z intake was partially missing, and there may have been recall bias. In addition, future studies are necessary to evaluate the value of the US FLI to explore the role of NAFLD in brain health. It may have significant research prospects.

## 5. Conclusions

Our results suggest that the risk of NAFLD might play a mediation role between higher dietary intake of L and Z, and cognitive functions, compared with the lowest intake, and that the risk of NAFLD may be an important intervention target in older adults. Therefore, early improvement in L and Z intake would be necessary to protect cognition by reducing the risk of NAFLD. More prospective studies and larger populations are needed to continue to verify the existence of the mediation effect and explore more pathways.

## Figures and Tables

**Table 1:** Characteristics of NHANES participants, 2011–2014.

Characteristic	All NHANES Participants Aged ≥ 60 Years (*n* = 3632)	NHANES Participants Included in this Analysis (*n* = 604)
Total (Weighted *n*)	59,784,355	29,397,912
NHANES cycle, *n* % (SE)		
2011–2012	49.06 (0.03)	51.11 (0.04)
2013–2014	50.94 (0.03)	48.89 (0.04)
Age (years), mean ± SE	69.40 ± 0.28	68.11 ± 0.32
Sex, *n*% (SE)		
Male	44.52 (0.01)	49.88 (0.02)
Female	55.48 (0.01)	50.12 (0.02)
Race/ethnicity, *n*% (SE)		
Mexican American	3.82 (0.01)	2.75 (0.01)
Other Hispanic	3.70 (0.01)	2.61 (0.01)
Non-Hispanic white	77.45 (0.02)	86.85 (0.02)
Non-Hispanic Black	8.99 (0.01)	5.39 (0.01)
Other/multiracial	6.05 (0.01)	2.40 (0.01)
Education >12 years, *n*% (SE)	80.87 (0.02)	87.76 (0.02)
GGT (IU/L), mean ± SE	26.39 (1.07)	24.90 (1.16)
Fasting insulin (pmol /L), mean ± SE	75.95 ± 2.81	75.40 ± 4.08
Waist circumference (cm), mean ± SE	102.76 ± 0.79	102.96 ± 0.95
Fasting glucose (mg/ dL), mean ± SE	112.77 ± 1.55	111.74 ± 1.80
Total energy intake (kcal), mean ± SE	1842.587 ± 27.57	1937.78 ± 34.93
Dietary L and Z intake (mg/day), mean ± SE	2091.91 ± 328.52	2592.69 ± 593.34
Total L and Z intake (mg/day), mean ± SE	2344.65 ± 353.85	2841.43 ± 644.91
CERAD W-L: Total Score (3 Recall trials), mean ± SE	19.82 ± 0.27	20.38 ± 0.27
Animal Fluency Test: Total Score, mean ± SE	17.96 ± 0.23	18.93 ± 0.27
DSST: Total Score, mean ± SE	51.71 ± 0.77	56.27 ± 0.92

Abbreviations: GGT: gamma glutamyltransferase; CERAD W-L: Consortium to Establish a Registry for Alzheimer’s disease Word Learning subset; DSST: the Digit Symbol Substitution Test.

**Table 2:** Mean (SD) scores on cognitive tests and US FLI, by quartile of L and Z intake (diet and total intake).

Group	Q1 (*n* = 152) Weighted (*n*) = 6, 469, 326	Q2 (*n* = 149) Weighted (*n*) = 6, 899, 307	Q3 (*n* = 152) Weighted (*n*) = 7, 924, 034	Q4 (*n* = 151) Weighted (*n*) = 8, 105, 244	*p*-Value for Difference
Dietary L and Z intake					
US FLI	31.43 (21.81)	34.46 (23.39)	29.19 (22.01)	23.48 (19.71)	**<0.001**
CERAD W-L: Total Score	18.70 (4.36)	19.46 (4.39)	19.60 (4.61)	20.39 (4.40)	**<0.05**
Animal Fluency Test: Total Score	16.74 (5.54)	17.56 (5.33)	18.07 (5.31)	18.81 (5.55)	**<0.05**
DSST: Total Score	46.36 (16.96)	49.49 (17.43)	52.11 (16.13)	54.01 (16.14)	**<0.001**
Z-score	−0.19 (0.79)	−0.05 (0.76)	0.05 (0.79)	0.19 (0.71)	**<0.001**
Total L and Z intake					
US FLI	31.82 (22.88)	34.14 (23.68)	28.60 (20.99)	23.95 (19.38)	**<0.001**
CERAD W-L: Total Score	18.56 (4.38)	19.49 (4.42)	19.75 (4.53)	20.35 (4.40)	**<0.05**
Animal Fluency Test: Total Score	16.46 (5.45)	17.96 (5.99)	18.09 (4.90)	18.68 (5.29)	**<0.05**
DSST: Total Score	45.14 (17.08)	49.84 (17.59)	52.88 (16.11)	54.11 (15.37)	**<0.001**
Z-score	−0.24 (0.79)	−0.04 (0.78)	0.09 (0.76)	0.19 (0.70)	**<0.001**

Continuous variables are expressed as means (standard error). *p*-values based on ANOVA for continuous variables, which accounted for National Health and Nutrition Examination Survey. Bold font indicates that the result is statistically significant.

**Table 3:** Associations of the US FLI and L and Z with cognitive function (β (95% CI)).

	CERAD W-L: Total Score	Animal Fluency Test: Total Score	DSST: Total Score	Z-Score
Crude model				
US FLI	−0.02 (−0.05, 0.00)	**−0.03 (−0.05, −0.01)**	**−0.10 (−0.20, −0.01)**	**−0.004 (−0.01, −0.00)**
Quartile of dietary L and Z				
Q2 vs. Q1	0.98 (−0.54, 2.51)	0.83 (−0.68, 2.34)	4.36 (−1.55, 10.26)	0.19 (−0.07, 0.45)
Q3 vs. Q1	1.22 (−0.10, 2.55)	1.01 (−0.29, 2.31)	5.34 (−0.40, 11.09)	**0.25 (0.02, 0.48)**
Q4 vs. Q1	**1.93 (0.41, 3.45)**	**2.64 (0.81, 4.47)**	**7.93 (2.27, 13.59)**	**0.42 (0.18, 0.66)**
Quartile of total L and Z				
Q2 vs. Q1	0.71 (−0.44, 1.85)	1.06 (-0.39, 2.50)	5.03 (−1.26, 11.32)	0.16 (−0.08, 0.40)
Q3 vs. Q1	**1.54 (0.45, 1.85)**	**1.85 (0.47, 3.22)**	**7.87 (2.17, 13.57)**	**0.36 (0.13, 0.60)**
Q4 vs. Q1	**1.88 (0.54, 3.22)**	**2.54 (0.91, 4.18)**	**8.93 (3.61, 14.25)**	**0.42 (0.21, 0.63)**
Adjusted model				
US FLI	−0.01 (−0.03, 0.01)	**−0.03 (−0.06, −0.01)**	**−0.09 (−0.16, −001)**	**−0.004 (−0.01, −0.00)**
Quartile of dietary L and Z				
Q2 vs. Q1	0.63 (−0.47, 1.72)	0.66 (−0.53, 1.85)	3.86 (−0.82, 8.55)	0.14 (−0.06, 0.33)
Q3 vs. Q1	1.07 (−0.01, 2.15)	0.72 (−0.49, 1.93)	4.28 (−0.48, 9.03)	**0.21 (0.03, 0.38)**
Q4 vs. Q1	**1.32 (0.05, 2.59)**	**2.15 (0.47, 3.84)**	**4.71 (0.60, 8.82)**	**0.29 (0.09, 0.49)**
Quartile of total L and Z				
Q2 vs. Q1	0.69 (−0.19, 1.57)	1.09 (−0.25, 2.44)	**4.87 (0.38, 9.35)**	0.16 (−0.02, 0.34)
Q3 vs. Q1	**1.42 (0.54, 2.31)**	**1.47 (0.22, 2.72)**	**6.13 (1.70, 10.57)**	**0.31 (0.13, 0.49)**
Q4 vs. Q1	**1.37 (0.19, 2.54)**	**2.10 (0.44, 3.76)**	**5.71 (1.58, 9.83)**	**0.30 (0.11, 0.49)**

Adjusted model: age, sex, race, the survey year, the total energy intake, the education level. Abbreviations: Q2, the second quartile; Q3, the third quartile; Q4, the fourth quartile; CERAD W-L, the CERAD Word Learning subtest; DSST, the Digit Symbol Substitution Test. Bold font indicates that the result is statistically significant.

**Table 4:** β (*p*) of the US FLI according to the quartile of dietary or total intake of L and Z.

	Q2 vs. Q1	Q3 vs. Q1	Q4 vs. Q1
	Quartile of dietary L and Z intake		
US FLI (Crude model)	1.22 (0.77)	−2.34 (0.61)	−10.99 (**<0.01**)
US FLI (Adjusted model)	0.74 (0.86)	−2.27 (0.61)	−10.23 (**<0.01**)
	Quartile of total L and Z intake		
US FLI (Crude model)	0.75 (0.85)	−4.48 (0.32)	−10.75 (**0.02**)
US FLI (Adjusted model)	0.43 (0.91)	−4.22 (0.32)	−9.84 (**0.02**)

Adjusted for age, race, sex, the survey year, the total energy intake, the education level. Bold font indicates that the result is statistically significant.

**Table 5:** Adjusted Model: Mediation Analysis of the relationship between quartile of dietary L and Z intake and cognitive function by the US FLI (β (95% CI)).

Relative Mediation Analysis	Q2 vs. Q1 ^1^	Q3 vs. Q1 ^1^	Q4 vs. Q1 ^1^
CERAD W-L: Total Score			
Direct Effect (c’_n_)	0.63 (−0.53, 1.76)	1.04 (−0.07, 2.17)	**1.21 (0.09, 2.37)**
Indirect Effect (a_n_ × b)	−0.01 (−0.16, 0.06)	0.03 (−0.03, 0.19)	0.11 (−0.03, 0.35)
PE (%)	-	-	-
Animal Fluency Test: Total Score			
Direct Effect (c’_n_)	0.68 (−0.77, 2.07)	0.65 (−0.76, 2.02)	**1.86 (0.31, 3.31)**
Indirect Effect (a_n_ × b)	−0.02 (−0.30, 0.18)	0.07 (−0.10, 0.35)	**0.30 (0.07, 0.75)**
PE (%)	-	-	**13.89**
DSST: Total Score			
Direct Effect (c’_n_)	3.93 (−0.11, 8.33)	**4.09 (0.20, 7.92)**	3.86 (−0.16, 7.97)
Indirect Effect (a_n_ × b)	−0.06 (−0.66, 0.59)	0.19 (−0.30, 0.98)	**0.84 (0.21, 1.95)**
PE (%)	-	-	**17.87**
Z-score			
Direct Effect (c’_n_)	0.14 (−0.05, 0.33)	**0.20 (0.01, 0.38)**	**0.25 (0.05, 0.44)**
Indirect Effect (a_n_ × b)	−0.01 (−0.03, 0.04)	0.01 (−0.01, 0.04)	**0.04 (0.01, 0.09)**
PE (%)	-	-	**13.79**

^1^ Adjusted model; age, sex, race, the survey year, the total energy intake, the education level. PE = relative indirect effect/ (relative direct effect + relative indirect effect). Statistically significant at no more than the 0.05 level. Bold font indicates that the result is statistically significant.

**Table 6:** Adjusted Model: Mediation analysis of the relationship between quartile of total L and Z intake (diet and supplement) and cognitive function by the US FLI (β (95% CI)).

Relative Mediation Analysis	Q2 vs. Q1 ^1^	Q3 vs. Q1 ^1^	Q4 vs. Q1 ^1^
CERAD W-L: Total Score			
Direct Effect (c’_n_)	0.70 (−0.40, 1.83)	**1.38 (0.27, 2.51)**	**1.26 (0.19, 2.43)**
Indirect Effect (a_n_ × b)	−0.004 (−0.14, 0.08)	0.05 (−0.02, 0.22)	0.11 (−0.03, 0.35)
PE (%)	-	-	-
Animal Fluency Test: Total Score			
Direct Effect (c’_n_)	1.11 (−0.41, 2.53)	**1.34 (0.05, 2.72)**	**1.80 (0.33, 3.19)**
Indirect Effect (a_n_ × b)	−0.01 (−0.30, 0.20)	0.13 (−0.04, 0.47)	**0.30 (0.07, 0.76)**
PE (%)	-	-	**14.29**
DSST: Total Score			
Direct Effect (c’_n_)	**4.90 (0.71, 9.34)**	**5.80 (1.66, 9.84)**	**4.92 (0.61, 9.18)**
Indirect Effect (a_n_ × b)	−0.03 (−0.60, 0.61)	0.34 (−0.11, 1.18)	**0.78 (0.18, 1.81)**
PE (%)	-	-	**13.68**
Z-score			
Direct Effect (c’_n_)	0.16 (−0.03, 0.36)	**0.30 (0.12, 0.49)**	**0.26 (0.07, 0.45)**
Indirect Effect (a_n_ × b)	−0.001 (−0.03, 0.02)	0.01 (−0.004, 0.05)	**0.03 (0.01, 0.09)**
PE (%)	-	-	**10.34**

^1^ Adjusted model; age, sex, race, the survey year, the total energy intake, the education level. PE = relative indirect effect/ (relative direct effect + relative indirect effect). Statistically significant at no more than the 0.05 level. Bold font indicates that the result is statistically significant.

## Data Availability

The data are available at https://www.cdc.gov/nchs/nhanes/index.htm (accessed date: 3 December 2021).
